# The Role of Work-Related Factors in the Development of Psychological Distress and Associated Mental Disorders: Differential Views of Human Resource Managers, Occupational Physicians, Primary Care Physicians and Psychotherapists in Germany

**DOI:** 10.3390/ijerph15030559

**Published:** 2018-03-20

**Authors:** Florian Junne, Martina Michaelis, Eva Rothermund, Felicitas Stuber, Harald Gündel, Stephan Zipfel, Monika A. Rieger

**Affiliations:** 1 Department of Psychosomatic Medicine and Psychotherapy, Medical University Hospital Tuebingen, University of Tuebingen, 72076 Tuebingen, Germany; felicitas.stuber@med.uni-tuebingen.de (F.S.); stephan.zipfel@med.uni-tuebingen.de (S.Z.); 2 Research Centre for Occupational and Social Medicine (FFAS), 79098 Freiburg, Germany; michaelis@ffas.de; 3 Institute of Occupational and Social Medicine and Health Services Research, University Hospital of Tuebingen, 72074 Tuebingen, Germany; Monika.Rieger@med.uni-tuebingen.de; 4 Department of Psychosomatic Medicine and Psychotherapy, University Hospital Ulm, University of Ulm, 89081 Ulm, Germany; eva.rothermund@uni-ulm.de (E.R.); harald.guendel@uniklinik-ulm.de (H.G.)

**Keywords:** work related stress, employees, occupational physicians, primary care physicians, psychotherapists, human resource managers

## Abstract

*Objectives*: This study analyses the perceived relevance of stress-dimensions in work-settings from the differential views of Human Resource Managers (HRM), Occupational Physicians (OP), Primary Care Physicians (PCP) and Psychotherapists (PT) in Germany. *Methods*: Cross-sectional study design, using a self-report questionnaire. Descriptive measures and explorative bivariate methods were applied for group-comparisons. Results are presented as rankings of perceived importance and as polarity profiles of contrasting views. *Results: N* = 627 participants completed the questionnaires (HRM: *n* = 172; OP: *n* = 133; PCP: *n* = 136; PT: *n* = 186). The stress dimensions with the highest mean ratings across all four professions were: *‘social relationships in the work place’* (*M* = 3.55, *SD* = 0.62) and *‘superiors´ leadership style’* (*M* = 3.54, *SD* = 0.64). Mean ratings of perceived relevance of stress dimensions differed most between HRM and the three medical professions. *Conclusions*: The perceived importance of work-related stress-dimensions seems to be higher in the medical disciplines (OP, PCP, PT) than in the group from the management sector (HRM). However, no fundamental disagreement on the role of work-related stress-dimensions seems to hinder e.g., intensified efforts of cooperation across sectors in tackling the “stress-pandemic” and improving the (mental) health of employees.

## 1. Introduction

Work-related psychological stress and stress-associated mental disorders cause a significant burden of disease and an extensive loss of quality of life lost in most industrial countries [1,2,3]. Current literature shows that mental disorders are one of the main reasons for years lived with disability (YLD) e.g., 175 millions years all over the world in 2010. Because of the pandemic character of mental disorders this constitutes a great challenge for health systems worldwide [4]. Furthermore Whiteford et al. emphasizes in their study that mental health has to be top priority in public health and prevention programs [4]. ‘Stress’ in general can be defined as a real or perceived threat to (physical, mental or emotional) homeostasis [5]. Despite the fact that psycho-social or mental distress may play a role in the onset or course of most mental disorders, entities that are commonly referred to as being potentially associated with work-related stress include adjustment disorders, anxiety disorders, ‘burn-out’ and (unipolar) depressive disorders or their respective symptoms [1]. The socio-economic consequences of (stress-related) mental disorders are significant due to, e.g., high sick-leave rates, long absences from work and high early-retirement rates [1,6,7].

Multiple studies have reported dimensions that may entail work related stress factors for employees. Findings of the current literature include stress inducing dimensions such as: quantitative job requirements [8,9,10], qualitative job requirements [8,11,12], work environment [13], organization of work processes [14,15], organization of working time [16], emotional demands in the work place [17,18,19], influence and development potential on the job [20,21], compatibility of family and work requirements [22], social relationships in the workplace [13,23,24], communication culture of the team/in the enterprise [23,25], managerial structure, superior’s leadership style [13,23,26] and the individual risk of employees [27,28,29,30].

As a result of the Whitehall II study, Virtanen et al. [9] for example found that within the dimension of *‘quantitative job requirements’* the factor ‘overwork’ was a predictor of major depressive disorder. With regards to the dimension of education or ‘*qualitative job requirements*’, Lunau et al. [11] presented findings that support the hypothesis that employees with lower levels of education experience higher stress-levels at work. The authors also inferred from their results that the policy context may be important in terms of e.g., an active labour market policy. Furthermore, participation rates in life-long-learning programs were interpreted as a protective factor by Lunau et al. [11]. Quantitative job demands and “underqualification” were also confirmed as predictors of cognitive complaints e.g., in a longitudinal study conducted in the Swedish context [8].

A well elaborated picture of relevant factors determining mental distress was presented by Finne et al. [23] indicating protective factors such as ‘*support of immediate superior*’ as well as ‘*fair leadership*’ as well as predictive factors such as ‘role conflict’ or ‘social climate’ in the work place. Another dimension investigated by Moen et al. [22] is the *‘work-family conflict’* which was identified by Moen and colleagues as a partial mediator between job demands and mental health outcomes of employees. The dimension of *‘organization of work processes’* was confirmed as a potential determinant of stress in different studies [14,15]. Concerning the dimension of *‘emotional demands at the work place’*, studies have shown the important role work-related emotional demands may play in the determination of psychological distress [17,19].

Given the accumulating evidence on potentially important psychological stressors in work-settings, preventive measures may be designed and implemented to reduce the burden of stress-associated mental disorders and to improve the quality of life (and productivity) of employees [31]. Among the most important professional stakeholders who may contribute to the development, implementation and practice of prevention and intervention programs tackling work-related stress and associated mental disorders are: Human Resource Managers (HRM), Occupational Physicians (OP), Primary Care Physicians (PCP) and Psychotherapists/Physicians of Psychosomatic Medicine (PT). HRMs may also be responsible for preventive programs and interventions in industrial companies (here in this context companies of the metal and electronic industries sectors), whereas OPs, PCPS and PTs may be associated with (secondary and tertiary) preventive programs and interventions because of their medical professions. The views and experiences of these profession groups can be seen as highly important perspectives that may well enrich the process of development of prevention and intervention measures [32,33].

The views and experiences of members of the above professions may e.g., be meaningful when selecting and prioritising specific dimensions that shall be addressed in e.g., prevention programs. Furthermore, if divergent views or attitudes towards work-related stressors prevail in different groups of stakeholders, they may act as barriers within newly designed (trans-sectorial) programs. Hence, it may be of vital importance to assess and to compare the views and experiences of HRMs, OPs, PCPs and PT regarding e.g., the differential importance of work-related stressors. Investigation of these groups may provide guidance e.g., for the selection of target dimensions and may ease the understanding of the different perspectives of involved stakeholders. 

This study therefore analyses the following research questions:

(1) What are the most important factors potentially associated with mental disorders in employees from the views of HRMs of industrial Companies, OPs, PCPs and PTs?
(2) What are the differential views of HRMs of Industrial Companies, OPs, PCPs and PTs concerning the relevance of specific work-related stressors in the development of (stress-associated) mental disorders?
(3) What are the differential views of HRMs of Industrial Companies, OPs, PCPs, and PTs concerning the relevance of the individual risks of employees in the development of (stress-associated) mental disorders?

The findings of this study aim to prioritise activities concerning prevention and intervention measures to tackle psychological (stress-related) symptoms and mental disorders in members of the industrial work force.

## 2. Study Population, Materials and Methods

### 2.1. Participants

A total of *N* = 627 participants completed the questionnaires (Human Resource Managers (HRMs): *n* = 172; Occupational Physicians (OPs): *n* = 133; Primary Care Physicians (PCPs): *n* = 136; Psychotherapists (PTs): *n* = 186) of which *n* = 303 (48.9%) were male and *n* = 317 (51.1%) were female participants with a total mean age of *M* = 52.8 years (*R* = 25–77). Seven participants did not supply information related to their gender, while 22 participants didn’t provide information concerning their age. All participants were situated in the German Federal State of Baden-Württemberg. Demographics and sample descriptions separated for all four occupational groups are depicted in Table 1 and Table 2.

The study was approved by the ethics commission of the medical faculty of the Eberhard Karls University Tuebingen (204/2014/BO2).

### 2.2. Study Design

The study was designed as an exploratory cross-sectional trial, based on a consistent self-report questionnaire for all four investigated groups of professions. The questionnaire was developed by the research team based on (a) qualitative research results [34,35,36] and (b) a literature search on the issue of work-related stress and its determinants see e.g., [37,38,39,40] e.g., as well as (c) structured group discussions within the multi-disciplinary study team (comprising experts from the fields of General Medicine, Occupational Medicine, Psychosomatic Medicine and Psychotherapy and Sociology), since no existing instrument that would sufficiently serve as the study aim was available. The questionnaire was piloted using “speaking out loud” methodology and structured feedback and consequently revised according to the pilot results. For the sake of transparency and completeness detailed results on all single items for all four groups are reported as part of the results section. The focus in the selection of dimensions and investigated factors can be seen in concordance with the Job-Demands-Resources-Model [41] and the dimensions in the German Copenhagen Psychosocial Questionnaire [37]). The potentially stress-determining factors and dimensions represented in the applied questionnaire are shown in Table 3.

The individual ratings of investigated factors were assessed by means of a four-point Likert scale (1 = not at all important, 2 = rather unimportant, 3 = rather important, 4 = very important). Piloting of the newly designed questionnaire within all four target groups included the dimensions feasibility, intelligibility, practicability and acceptance. 

Distribution of questionnaires was organised via postal mailings to potential participants. Questionnaires were sent to: 1000 randomly selected PCPs and 700 randomly selected out-patient PTs (accessed via public internet-based registers respectively), 450 OPs and 1426 HRMs (complete sample), (accessed via professional associations respectively), all situated in the German Federal State of Baden-Württemberg. Following the initial hard copy mailing of questionnaires, one hard copy post-card reminder was sent out to all potential participants 14 days later.

Response rates were as follows: HRMs 12% (*n* = 172), OPs 30% (*n* = 133), PCPs 14% (*n* = 136) and PTs 27% (*n* = 186). Non-responder analyses showed that female Ops responded more often than male OPs. In the group of PCPs response rates in smaller cities were higher than in larger cities. HRMs in smaller industrial companies were more likely to respond than their colleagues in larger companies [42].

### 2.3. Statistical Analyses

Statistical analyses of the Likert-scaled items and scores included descriptive measures in terms of means (*M*) and measures of dispersion (standard deviation (*SD*)). Explorative bivariate methods were applied when comparing e.g., different profession groups using the Mann-Whitney-U test for independent ordinal data and metric data in the case of missing normal distribution.

We considered Bonferroni-adjustments for levels of significance in the case of multiple testing on the bivariate level between groups. Effect sizes for Mann-Whitney-U- and Wilcoxon tests were calculated as ‘*w*’ (test value/√number of cases) and categorised as follows: low <0.3, moderate 0.3–0.5, high: >0.5 [43]. Due to satisfying completeness of questionnaires (usually less than 10% missing data), no imputation measures were considered. Besides single item comparisons, sum-scores were used for the analyses of group differences. Sum-scores were calculated along content dimensions of included items. See Table 3 for the explanations and grouping of items within sum-scores. The items *‘work environment’* and *‘individual risks of employees’* could not be included in one of the sum scores for content reasons and are tested on a single item. Further details on the study design and applied statistical methods are published elsewhere [42]. With regards to the terminology used in this manuscript, the term “factor” describes specific single factors and is used with the questionnaire single items. The term “dimension” is used when several single factors can be categorized in a common perspective such as for example “work environment”. The organization of several factors under a common dimension to ease understanding and interpretation does not imply that the term dimension refers to an established psychological construct.

## 3. Results

With regards to study question (1) it can be summarised, that all four groups in general saw the importance of the investigated work-related stressors. The three factors with the highest ratings across all four professions were: 1. *‘social relationships in the work place’* (*M* = 3.55, *SD* = 0.62); 2. *‘superior’s leadership style’* (*M* = 3.54, *SD* = 0.64) and 3. *‘quantitative job requirements’* (*M* = 3.43, *SD* = 0.66). However, the different groups showed different rankings of importance for the investigated specific factors.

### 3.1. Rankings of Importance of Potential Stress-Dimensions by Group of Profession

The most important specific factor among the investigated items according to the group of Occupational Physicians (OPs) was the *‘superior’s leadership style’* (*M* = 3.68; *SD* = 0.55), whereas the least important factor according to OPs was the *‘qualitative working requirements’* (*M* = 2.97; *SD* = 0.76). For Primary Care Physicians (PCPs) the most important factor among the investigated set of factors was *‘social relationships in the work-place’* (*M* = 3.69; *SD* = 0.49) and the least important factor in this group was the *‘working environment’* (*M* = 2.72; *SD* = 0.82). 

For Psychotherapists (PTs), also the factor *‘social relationships in the work-place’* was the most important single item (*M* = 3.74; *SD* = 0.48) whereas ‘*qualitative working requirements*’ (*M* = 2.77; *SD* = 0.73) and ‘*working environment*’ (*M* = 2.77; *SD* = 0.75) were seen by PTs as the least important factors investigated here.

Human Resource Managers (HRMs seem to agree with PCPs and PTs with regards to the most important factor of *‘social relationships in the work-place’* (*M* = 3.28; *SD* = 0.73) but absolute values for importance are almost consistently lower for most work-related factors in the group of HRMs. The least important factor from the perspective of HRMs among the investigated items was the ‘*working environment*’ (*M* = 2.45; *SD* = 0.68). See Table 4 and Table 5 for further details on the dimension and factor ranking of stressors.

### 3.2. Sum-Score Group Differences of Perceived Relevance of Stress-Dimensions

With regards to overall trends concerning the importance of work-related stressors between the four groups, it was found that HRMs consistently rated the importance of work-related factors lower than the three “medical” groups, i.e., OPs, PCPs and PTs.

The largest (bivariate) group-differences were found for the dimension sum score ‘*Content of work*’ (items 1, 2, 6, 7) between the groups of HRMs and PTs with a moderate effect size (*w* = 0.39, *p* < 0.001, *n* = 354). The largest (bivariate) group-difference for the dimension sum score ‘*organization of work processes*’ (items 4, 5, 8) with moderate effect size was again found between the groups of HRMs and PTs (*w* = 0.44, *p* < 0.001, *n* = 354) as well as for the dimension sum score *‘social relationships in the work-place’* (items 9–12) (*w* = 0.44; *p* < 0.001, *n* = 354). Group differences with moderate effect size were also found between the groups HRMs and OPs. The dimensions ‘*Content of work*’ (*w* = 0.32, *p* < 0.001, *n* = 303), ‘*organization of work processes*’ (*w* = 0.30; *p* < 0.001, *n* = 303), as well as the dimension *‘social relationships at the work-place’* (*w* = 0.38; *p* < 0.001, *n* = 303), differ all significantly*.* Furthermore the sum scores for all three dimensions *Content of work*’ (*w* = 0.34, *p* < 0.001, *n* = 306), ‘*organization of work processes*’ (*w* = 0.40; *p* < 0.001, *n* = 306) and *‘social relationships in the work-place’* (*w* = 0.40, *p* < 0.001, *n* = 306) show significant differed with moderate effect size for the occupational groups HRM and PCP. A significant group difference was also found in between the groups of medical professions (PCP, OP, PT). The dimension ‘*organization of work processes*’ showed a group difference between OPs and PTs but with a low effect size (*w* = 0.15, *p* < 0.001, *n* = 317) (see Table 4 and Table 5). For statistical group tests on single factor item level see Supplementary Materials.

### 3.3. Relative Group Differences of Perceived Importance of Specific Stressors

Two general trends with regards to relative appraisal of the importance of stressors were found in the data when testing for group-differences. The largest difference across most of the investigated factors was found between the “medical group” (OP, PCP, PT) and the “management” profession of HRMs. Another general trend can be seen between the profession groups working within the industrial sector (HRM and OP) and the two professions working outside the industrial context (PCP and PT). The single items with the largest relative differences between HRMs and one of the professions of the “medical group” included the factor *‘organization of working time’* for the comparison of HRM vs. PCP, (*w* = 0.46; *p* < 0.001) and for the comparison HRM vs. PT (*w* = 0.45; *p* < 0.001).

**Figure 1:**
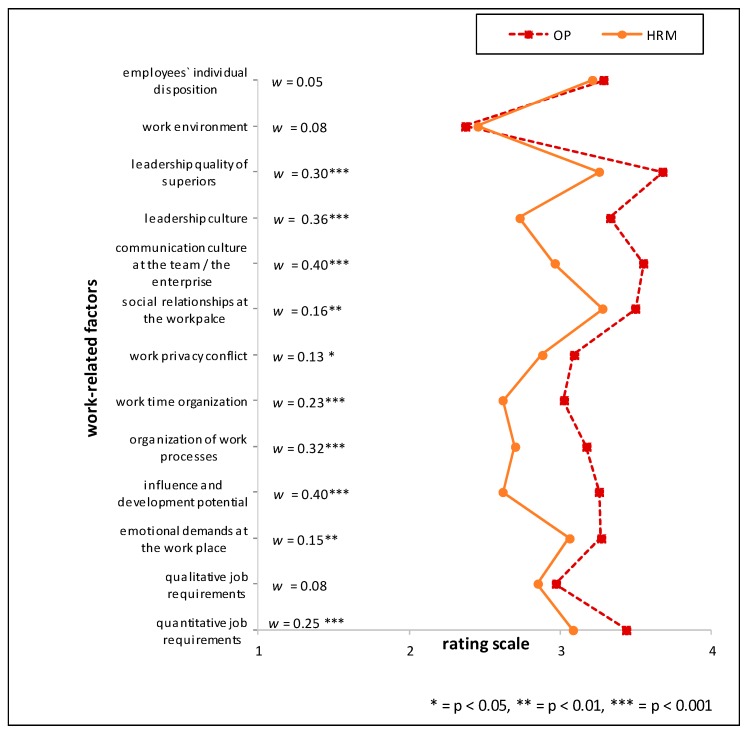
Polarity profile across work-related factors potentially important for (stress-associated) mental disorders in employees comparing the mean value ratings from “not at all important” (1) to “very important” (4) of Human Resource Managers (HRM) and Occupational Physicians (OP), w = effect size of differences.

**Figure 2:**
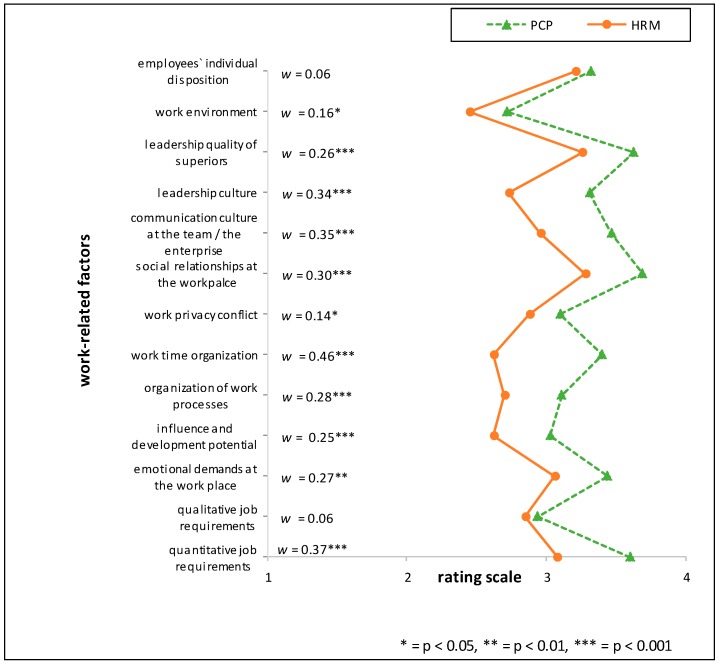
Polarity profile across work-related factors potentially important for (stress-associated) mental disorders in employees comparing the mean value ratings from “not at all important” (1) to “very important” (4) of Human Resource Managers (HRM) and Primary Care Physicians (PCP), w = effect size of differences.

**Figure 3:**
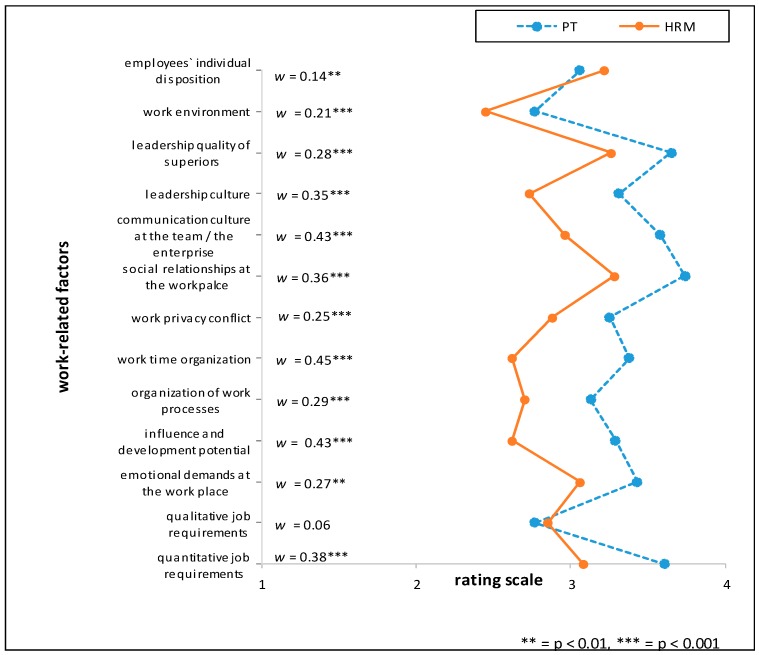
Polarity profile across work-related factors potentially important for (stress-associated) mental disorders in employees comparing the mean value ratings from “not at all important” (1) to “very important” (4) of Human Resource Managers (HRM) and Psychotherapists (PT), w = effect size of differences.

**Figure 4:**
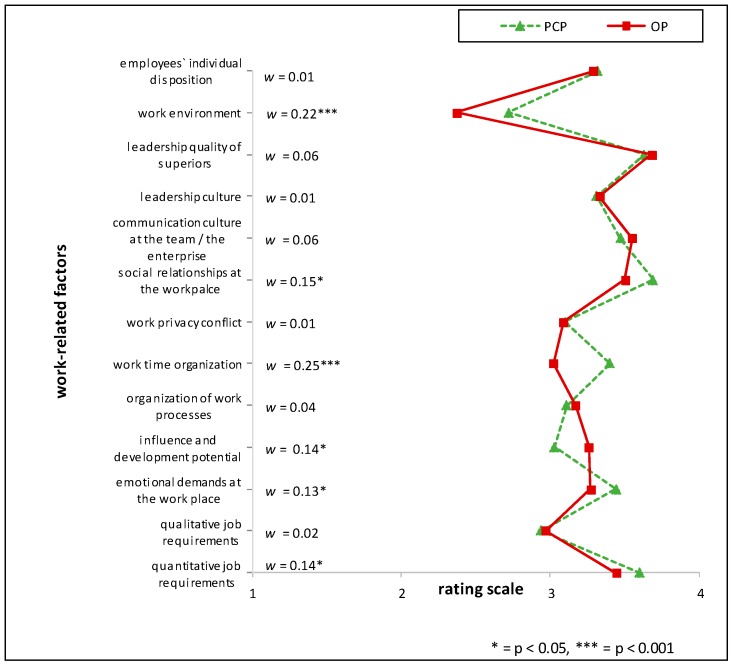
Polarity profile across work-related factors potentially important for (stress-associated) mental disorders in employees comparing the mean value ratings from “not at all important” (1) to “very important” (4) of Occupational Physicians (OP) and Primary Care Physicians (PCP), w = effect size of differences.

**Figure 5:**
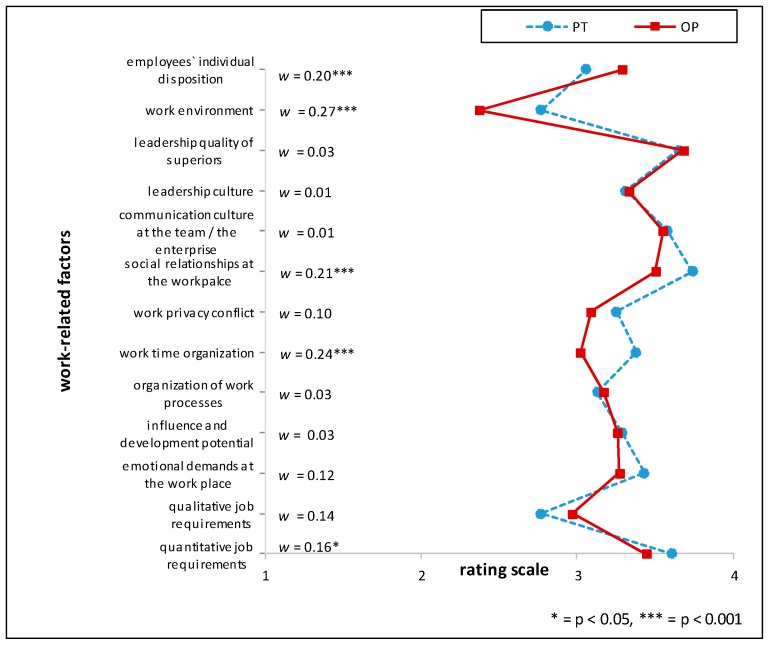
Polarity profile across work-related factors potentially important for (stress-associated) mental disorders in employees comparing the mean value ratings from “not at all important” (1) to “very important” (4) of Occupational Physicians (OP) and Psychotherapists (PT), w = effect size of differences.

**Figure 6:**
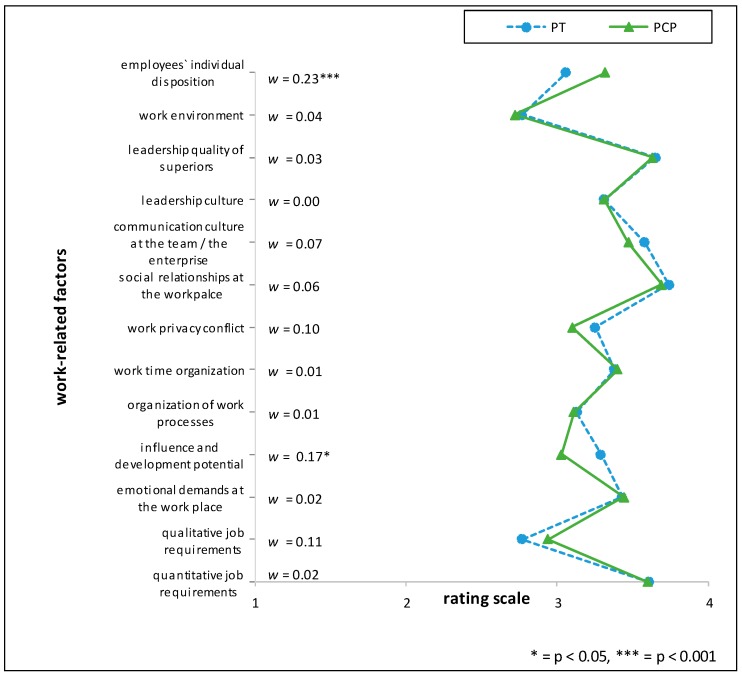
Polarity profile across work-related factors potentially important for (stress-associated) mental disorders in employees comparing the mean value ratings from “not at all important” (1) to “very important” (4) of Primary Care Physicians (PCP) and Psychotherapists (PT), w = effect size of differences.

Comparing HRM with PT the items ‘*influence and development potential on the job*’ (mean rating PT > HRM, RD = 26%), *‘communication culture of the team/in the enterprise’* and *‘managerial structure’* (mean rating PT > HRM, RD = 21% respectively) showed the highest relative differences. Between the two professions situated in the industrial context (HRM and OP), the largest relative difference (RD) was found when comparing ‘*influence and development potential on the job*’ (mean rating OP > HRM, RD = 20%). Further marked differences in the appraisal of importance between the HRM and OP groups were *‘managerial structure’* (mean rating OP > HRM, RD = 18%) and the *‘communication culture of the team*/*in the enterprise’* (mean rating OP > HRM, RD = 17%) (see Figure 1, Figure 2 and Figure 3 and Table 6). For effect sizes of group comparisons between the groups PT, OP, PCP, see Figure 4, Figure 5 and Figure 6.

(Please note that lines in the graphs of Figure 1, Figure 2, Figure 3, Figure 4, Figure 5 and Figure 6 does not imply any mathematical relationship of single factors).

### 3.4. Relative Group Differences of Importance of ‘Individual Risks’ of Employees

Overall, the importance of employees’ individual risks was rated similar compared to the work-place related factors in terms of absolute mean values (*Mean Range*: 3.06–3.29). However, PTs saw the individual risk less important than the three other groups in terms of e.g., the frequency they choose “very important” as the answer to the question: “how important is the individual risk of the employee in the development of (stress-related) mental disorders?” (PTs = 18% (*n* = 32); OPs 34.8% (*n* = 46); PCPs 33.8% (*n* = 46); HRMs 33.3% (*n* = 56). Significant group differences for the item ‘*individual risk of employees*’, albeit with low effect sizes, were found between OPs and PTs (*w* = 0.20, *p* < 0.001, *n* = 308) and when comparing PTs and PCPs (*w* = 0.23, *p* < 0.001, *n* = 312) (see Supplementary Materials).

## 4. Discussion

To the best of our knowledge, this is the first study investigating the (differential) perceptions of the importance of work-related stressors together with the individual risk of employees in the development of (stress-associated) mental disorders in employees from the perspective of the four professional groups Human Resource Managers (HRMs), Occupational Physicians (OPs), Primary Care Physicians (PCPs) and Psychotherapists (PTs). The results of this study show, that all four groups do see the importance of the investigated work-related stressors as well as the individual risk of employees as determinants in the development of (stress-related) mental disorders.

However, the results also show that the ratings by HRMs for the importance of work-related stressors are markedly lower than the ratings of the three ‘medical groups’ (OP, PCP, PT). In turn, the importance of individual risk is seen more important by HRMs, OPs, PCPs than by the group of PTs. Hence, the data of this study implicate different perceptions when comparing e.g., the ‘non-medical’ (HRM) to the ‘medical’ professions (OP, PCP, PT). Another general trend within the data of this study can be seen in differences in the perception of the importance of factors between the professions from *within* the industrial context (HRM, OP) and the medical professions working *outside* the industrial sector (PCP, PT). The latter professions thereby tend to value the work-related stressors higher.

The three factors with the highest ratings across all four professions were: ‘*social relationships in the work place*’; ‘*superiors leadership style*’ and ‘*quantitative job requirements*’. These findings are in line with the results of previous studies. For example, Tsuno et al. [24] identified “social relationships in the work place” for the stress dimension and stated that “intragroup conflict” was associated with greater psychological distress for males. For the stress dimension “superiors leadership style” Finne et al. showed that in 48 organizations with many different professions “support from immediate superior” and “fair leadership” were two of the three most protective dimensions for subordinates in terms of mental health problems [23]. Furthermore, as a cross sectional study from the Swedish context showed, high quantitative demands were associated with cognitive complaints (e.g., memory dysfunction) [8]. The ranking of factors across all four groups (together with the individual rankings of each group) thereby supports the idea of the social or rather *interpersonal and interactional* dimension of work-place realities as the most important category in the development of psychological stress and stress associated mental disorders in employees. Hence, the “human dimension” of interpersonal/interactional competencies can be supported by the results of this study as a potentially important target within primary, secondary and tertiary prevention measures for employees and patients suffering from stress-related mental disorders [13,23,24,25,26]. Current reviews of the related literature seem to confirm the influence of social aspects in the workplace on stress-levels and associated disorders in employees, although the effects of “missing social support” or “bad leadership quality” e.g., on the development of symptoms of depression or 'burnout' is in some studies were found to be rather small or moderate [46,47,48]. However, Theorell et al. [46] were able to show that bullying as one part of an interpersonal dimension has a significant impact on the development of depression symptoms. A relatively small but increased risk was shown for interpersonal relationships in the workplace descripted in the metaanalytic study by Stansfeld et al. [48]. However, studies applying longitudinal designs on the matter are limited.

The role of the ‘*superior’s leadership style*’ as another important aspect of the interpersonal dimension may thereby be seen as of even more importance from the perspective of prevention initiatives, since e.g., the superior’s example and multiplication role may be of significant importance for the interpersonal and interactional culture of a working team or group [13,23,26]. One theoretical concept that addresses the influence of the (group-)leader on interpersonal dimensions is the concept of the Transformational Leadership [49]. This leadership style was shown to positively influence the attitude, motivation and well-being of employees by using for example “inspirational motivation”, “intellectual stimulation”, and “individualized consideration as leadership techniques [49,50]. Franke and Felfe [51] showed in their study that all dimensions of transformational leadership are associated with reduced job strain of employees. These findings seem to support the findings of the here presented study in the sense that “superior’s leadership style” may represent an important potential perspective of prevention.

The main focus of the here presented study was work-related factors as opposed to the individual predisposing factors for perceived stress and (stress-related) mental disorders of employees. For example the personality factors of employees were represented only indirectly by the global item ‘*individual risks of employees’* and hence, it cannot be inferred from the results whether different personality characteristics such as e.g., ‘conscientious’/’compulsive’ or ‘impulsive’ personality traits or ‘extraversion’ and ‘openness’ may be seen as more or less important components of the individual risk of employees from the perspective of the investigated professional groups. However, other studies have already investigated the association between personality and perceived work stress. For instance Burgess et al. found no significant positive association between personality and workplace stress in a sample of intensive care unit nurses. The results of the study by Burgess et al. however, showed an association between ‘openness’ and ‘extraversion’ with the handling of difficult patients and relatives in the sense that participants with high levels of openness and extraversion show a lower level of stress in dealing with difficult patients and their dependents [52].

The rankings of the most important and the least important factors within each of the four professional groups show different patterns. With regards to the single most important factor within each of the four groups the factor ‘*social relationships in the workplace*’ turned out to be the most important factor in the group of HRMs, PCPs and PTs, whereas OPs rated the ‘*superior’s leadership style*’ as the most important factor within the investigated set of dimensions with potential stressors. On the other hand, the least important factor determining stress from the view of OPs and PTs was ‘*qualitative job requirements*’. For the professional groups HRMs and PCPs the lowest ratings were found for the factor ‘*work environment*’.

The last two findings may be particularly surprising given that the qualitative job requirements and the work environment (e.g., noise, dirt, hazardous substances) can indeed be assumed to be a significant and fundamental factor that eventually may cause substantial distress. With regards to qualitative job requirement, as shown in the studies of Stenfors et al., low qualification had a positive relation with cognitive complaints [8]. Furthermore, Lunau et al. investigated the association between educational attainment and work stress in workers of 16 different European countries. They found that in every country participants with a low educational level perceive more stress at work. These results may support the hypothesis of a “social gradient of work stress” [11]. This notion is also supported by the results of Rugulies et al. In a randomized and stratified sample of Danes the authors report that unemployed or working but unskilled Danish participants showed a higher prevalence of depressive symptoms compared to the group of higher-grade academics [12]. With regards to the work environment, Applebaum et al. evaluated a sample of over one hundred medical-surgical nurses working in the setting of acute care. The factor ‘noise’ was thereby significantly associated with perceived stress. This result emphasizes the assumption that environmental conditions can indeed be stressful to clinical staff [53]. However, the low ratings for the dimension of ‘work environment’ may be due to the existing high standards of occupational safety regulations regarding the work environment in Germany. These standards may lead to the impression that the “human dimensions” are relatively more important to the majority of employees who have participated in this survey. The successful history in improving the dimension of the work environment within the German industrial sector, might lead to the rather optimistic position that, similarly effective standards regarding e.g., stress-preventing leadership styles or interpersonal team-cultures may be possible to establish. However, standards regarding these dimensions indeed are far more complex to define and to monitor, than e.g., the standards of a certain maximum of e.g., the decibel level within a working environment. Nevertheless a first attempt to formalise the evaluation of psychological hazards in the occupational context is the risk assessment of the newly established ‘work conditions act’ of the Federal Republic of Germany. This new legislation comprises several “human dimensions” e.g., “communication and cooperation” or “social interaction with colleagues and superiors´” as dimensions that may entail potential risk factors associated with the mental health of employees that need to be monitored by companies [38].

The overall consensus of the four professional groups on the importance of the investigated dimensions of potential work-related stress can be seen as a promising finding towards possible agreements on the general necessity and the direction of stress-prevention measures and improved structures of (health) services in this domain. However, as the data of this study imply, the importance of the sense of urgency for improvement seems to be higher in the groups of the medical realm (OP, PCP, PT) than in the management sector (HRM). Indeed these differences may be due to a “frame of reference bias” given that employees who are affected by stress-associated symptoms or disorders present to the medical groups rather than in HRMs. Furthermore, the rating of HRMs may be influenced in their ratings by a more defensive attitude towards the working context they are (in parts) responsible for, or HRMs may tend to “externalize” the relevant factors from the working context more toward the individual employee. In turn, the members of the medical professions may indeed be more likely to “externalize” results from the individual client/patient they care for to the working context. On the other hand, one could argue, that the members of the medical professions including psychotherapists, do have the more detailed experience and professional insights e.g., into the causes, onsets and courses of mental health impairments and hence, they may be the more reliable professions when the relative importance of the investigated dimensions is in question. Despite these different perspectives, it can be inferred from the findings of this study, that the importance and the related ‘sense of urgency’ for the implementation or improvement of stress-prevention measures for employees seems to be lower within the management discipline of HRMs than in the medical-related professions. This finding may call for a more active part of the industry itself and indeed for more cooperation and exchange of experiences and ideas of the different professional stakeholders.

Besides the relative differences in terms of the perceived importance of the work-related stress-dimensions, this study showed that there are no substantial barriers to intensified cooperation among the investigated professions at least in terms of missing fundamentally opposing views regarding the here investigated dimensions.

## 5. Limitations

The investigation was focused on the geographical area of the German Federal State of Baden-Württemberg including participants from rural, peripheral urban and urban contexts. HRMs and OPs were recruited from the complete size-spectrum of companies. With regards to the latter distributions (see also Table 2) we are confident that the direction of the findings may be consistent with the German context at large and that the cohorts are representative of the four professional groups. 

With regards to the Country-specific aspects of the results of this study, Shackelton et al. for example compared stress levels in employees between the US, the UK and Germany. Here the highest stress levels were found in German workers [54]. Similarly, in a comparison with Australian workers also German employees also showed higher stress levels [55]. Hence, if levels of perceived stress differ across countries, one should compare the perceptions of stress-causation with great caution and the results of this study from the German context are not easily transferable to other countries and cultural contexts. However, as Salavecz et al. showed that the association of for example (a) the effort-reward imbalance at work or (b) over commitment at work, with poor self rated health was seen in their international comparison of European countries, was present in all investigated countries. Hence, the mechanisms at work seem comparable in parts, but the explanation of variance by different determinants may vary considerably across contexts. Hence, it may be of huge interest to the field to replicate this study by comparing different cultural contexts and countries [56].

The results of the non-responder analyses can be interpreted as reassuring that no systematic bias may fundamentally impair the data and the results of this study. Given the cross-sectional nature and due to the nature of the research questions no causal relationships with regards to the role of stress dimensions in the genesis of stress and stress associated diseases can be inferred from the results.

## 6. Conclusions

This is the first study investigating the perceived relevance of a broad range of potential work-related stressors in the genesis of (stress-related) mental health impairment in employees from the perspective of HRMs, OPs, PCPs and PTs. The results imply that from the perspective of the investigated groups the factors ‘*social relationships in the workplace’* and ‘*superior’s leadership style*’ are seen as the most relevant single dimensions among the investigated set of dimensions ultimately responsible for causing work related distress of employees. The findings of this study may potentially inform efforts of prioritising targets within stress-prevention and -intervention programs Targeted intervention measures may in general well be necessary to tackle the ‘stress-pandemic’ and to ultimately reduce the burden of stress-associated mental health impairment and improve the quality of life (and productivity) of employees.

## Figures and Tables

**Table 1:** Demographic characteristics of the study sample.

Variable	HRM		OP		PCP		PT	
	%	Total	%	Total	%	Total	%	Total
Sex								
Male	58.2	96/165	52.6	70/133	59.6	81/136	30.1	56/186
Qualification of the PCP group								
Specialist title	-	-	54.9	73/133	76.1	102/134	35.5	66/186
Status of the investigated OP								
Employed in the enterprise	-	-	46.0	54/128	-	-	-	-
Employed in an external occupational health service	-	-	24.2	31/128	-	-	-	-
Own practice	-	-	28.9	37/128	-	-	-	-
Freelance without own practice	-	-	4.7	6/128	-	-	-	-
Size of enterprise								
Large (≥250 employees)	53.6	89/166	93.9	108/115	-	-	-	-
Medium-sized (50≥ and <250)	38.0	63/166	4.3	5/115	-	-	-	-
Small (<50)	8.4	14/166	1.7	2/115	-	-	-	-
Location of the OP/PCP practice								
City	-	-	57.0	73/128	30.9	42/136	-	-
City periphery	-	-	27.3	35/128	37.5	51/136	-	-
Countryside	-	-	15.6	20/128	31.6	43/136	-	-
Status of HRM in the enterprise								
Executive director /owner	17.5	28/160	-	-	-	-	-	-
Human resource managers	75.0	120/160	-	-	-	-	-	-
Other	7.5	12/160	-	-	-	-	-	-

*Note*: Abbreviations: HRM = Human Resource Managers (*n* = 172), OP = Occupational Physicians (*n* = 133), PCP = Primary Care Physicians (*n* = 136), PT = Psychotherapists (*n* = 186), % = percent (*n*_(applied)_/*n*_(total,valid)_).

**Table 2:** Age distribution of subsamples.

Variable	*n*	*M*	*SD*	*Min–Max Score*
Occupational physician (OP)	133	54.9	8.0	36–77
Primary care physicians ^a^ (PCP)	130	53.7	8.6	37–75
Psychotherapist (PT)	183	53.9	8.6	31–71
Human resource managers (HRM)	159	48.8	8.1	25–66

*Note*: ^a^ The PCP sample included *n* = 7 participants who were certified specialists for occupational health.

**Table 3:** Items and dimensions of applied questionnaire.

Dimensions	Individual Factor Items	Examples Given in the Questionnaire	Source
Work contents (4 items)			
1a	Quantitative job demands	Quantitative amount of the employee’s tasks	[37]
1b	Qualitative job demands	Content of the employee’s work and the level of expertise/competencies/education/intellectual capacity required	[37]
1c	Emotional demands in the workplace	Exposure to emotionally stressful events/work phases or emotional dimension of accountability/responsibility (e.g., during management of entrepreneurial crises)	[37]
1d	Influence and development potential on the job	How work processes can be influenced/shaped by the executing employee and how the employee has the opportunity for personal growth/development within his work setting/processes.	[37]
Organization of work processes (3 items)			
2a	Organization of work processes	Description of work processes and definitions e.g., in terms of who is involved. transparency of duties within the team along the work processes etc.	[44]
2b	Working time organization	Shift work, duration of daily working time	[39]
2c	Work-privacy conflict	Structural dimension of compatibility of family and working life e.g., in terms of child care offerings, forecast reliability of absence from work etc.	[37]
Interpersonal relations and leadership (4 items)			
3a	Social relationships in the workplace	Working atmosphere in a team/ in the enterprise	[37]
3b	Communication culture in the team/in the enterprise	Formal and informal ways and styles of communication among team members and within the enterprise at whole (transparency, timeliness, implicit/explicit ways of communication) etc.	^a^
3c	Leadership culture	Hierarchies (flat/steep), accountabilities, accessibility etc.	[40]
3d	Leadership quality of superiors	Leadership quality including interpersonal competencies of superiors	[37]
Physical work environment			
4a	Physical work environment	Structural dimension including e.g., noise exposure, lighting conditions, cleanliness, workplace ergonomics	[45]
	(Individual level)		
Individual disposition			
5a	Individual disposition of employee	The individual disposition of an employee in terms of e.g., stress-resilience, individual resources, prehistory of common mental disorders etc.	^a^

*Note*: ^a^ General item for individual disposition.

**Table 4:** Perceived relevance of work-related stress-dimensions.

Stress-Dimensions	HRM		OP		PCP		PT	
	*M*	*SD*	*M*	*SD*	*M*	*SD*	*M*	*SD*
1. Work content	2.91	0.49	3.23	0.45	3.25	0.46	3.28	0.39
2. Work organization	2.73	0.58	3.09	0.53	3.21	0.50	3.25	0.49
3. Interpersonal relations and leadership at work	3.06	0.61	3.51	0.49	3.53	0.45	3.57	0.43

*Note*: Abbreviations: HRM = Human Resource Managers (*n* = 170), OP = Occupational Physicians (*n* = 133), PCP = Primary Care Physicians (*n* = 136), PT = Psychotherapists (*n* = 184), 1 = not important at all, 2 = rather unimportant, 3 = rather important, 4 = very important.

**Table 5:** Most important and least important work-related factors for the development of (stress-associated) mental disorders according to the ratings of the investigated groups of professions.

Factors with Highest Rankings				Factors with Lowest Rankings				
Position	Item	*M*	*SD*	Position	Item	*M*	*SD*	*n*
Human Resource Managers								
1	Social relationships in the workplace	3.28	0.73	12	Physical work environment	2.45	0.68	170
2	Leadership quality of superiors	3.26	0.75	11	Working time organization	2.62	0.84	168
3	Quantitative job demands	3.08	0.72	10	Influence and development potential on the job	2.62	0.78	169
Occupational Physicians								
1	Leadership quality of superiors	3.68	0.55	12	Qualitative job demands	2.97	0.76	127
2	Communication culture in the team/in the enterprise	3.55	0.60	11	Physical work environment	2.37	0.73	133
3	Social relationships in the workplace	3.50	0.64	10	Working time organization	3.02	0.78	132
Primary Care Physicians								
1	Social relationships in the workplace	3.69	0.49	12	Physical work environment	2.72	0.82	136
2	Leadership quality of superiors	3.63	0.54	11	Qualitative job demands	2.94	0.80	135
3	Quantitative job demands	3.60	0.55	10	Work-privacy conflict	3.10	0.75	136
Psychotherapists								
1	Social relationships in the workplace	3.74	0.48	12	Qualitative job demands	2.77	0.73	184
2	Leadership quality of superiors	3.65	0.56	11	Physical work environment	2.77	0.75	181
3	Communication culture in the team/in the enterprise	3.58	0.54	10	Organization of work processes	3.13	0.66	184

*Note*: Abbreviations: HRM = Human Resource Managers, OP = Occupational Physicians, PCP = Primary Care Physicians, PT = Psychotherapists.

**Table 6:** Largest group differences between HRM and the three medical professions.

Factors with the Largest Group Differences between HRM and Medical Groups		
Ranking Position	Items	w
HRM vs. OP		
1	communication culture at the team/the enterprise	0.40
2	influence and development potential	0.40
3	leadership culture	0.36
HRM vs. PCP		
1	work time organization	0.46
2	quantitative job requirements	0.37
3	communication culture of the team/the enterprise	0.35
HRM vs. PT		
1	work time organization	0.45
2	influence and development potential	0.43
3	communication culture of the team/ the enterprise	0.43

*Note*: Abbreviations: HRM = Human Resource Managers, OP = Occupational Physicians, PCP = Primary Care Physicians, PT = Psychotherapists, *w* = effect size of differences.

## Supplementary Materials

The following are available online at http://www.mdpi.com/1660-4601/15/3/559/s1, Table S1: Mean values and statistical group tests—single item level.
