# Supplementary material for: The Role of Work-Related Factors in the Development of Psychological Distress and Associated Mental Disorders: Differential Views of Human Resource Managers, Occupational Physicians, Primary Care Physicians and Psychotherapists in Germany
Source: Int J Environ Res Public Health. 2018 Mar 20;15(3):559. doi: 10.3390/ijerph15030559 (PMC5877104; doi:10.3390/ijerph15030559)
Supplement: Supplementary file 1 [file ijerph-15-00559-s001.pdf]

Supplementary Materials

Table S1. Mean values and statistical group tests – single item level.

Dimension	Items	HRM		OP		PCP		PT		N		HRM-OP	HRM-PCP	HRM-PT	OP-PCP	OP-PT	PT-PCP
		M	*SD*	*M*	*SD*	*M*	*SD*	*M*	*SD*	*M*	*SD*	*w*	*w*	*w*	*w*	*w*	*w*
1 Work contents	1a Quantitative job demands	3.08	0.72	3.44	0.60	3.60	0.55	3.61	0.58	3.43	0.66	***0.25	***0.37	***0.38	*0.14	*0.16	0.02
	1b Qualitative job demands	2.85	0.76	2.97	0.76	2.94	0.80	2.77	0.73	2.87	0.76	0.08	0.06	0.06	0.02	0.14	0.11
	1c Emotional demands at the workplace	3.06	0.72	3.27	0.66	3.44	0.66	3.43	0.62	3.30	0.68	**0.15	***0.27	***0.27	*0.13	0.12	0.02
	1d Influence and development potential on the job	2.62	0.78	3.26	0.66	3.03	0.79	3.29	0.66	3.04	0.77	***0.40	***0.25	***0.43	*0.14	0.03	*0.17
2 Work organization	2a Organization of work processes	2.70	0.75	3.17	0.62	3.11	0.70	3.13	0.66	3.02	0.71	***0.32	***0.28	***0.29	0.04	0.03	0.01
	2b Working time organization	2.62	0.84	3.02	0.78	3.40	0.64	3.38	0.68	3.10	0.81	***0.23	***0.46	***0.45	***0.25	***0.24	0.01
	2c Work privacy conflict	2.88	0.73	3.09	0.76	3.10	0.75	3.25	0.70	3.08	0.74	*0.13	*0.14	***0.25	0.01	0.10	0.10
3 Interpersonal relations and leadership at work	3a Social relationships at the workplace	3.28	0.73	3.50	0.64	3.69	0.49	3.74	0.48	3.55	0.62	**0.16	***0.30	***0.36	*0.15	***0.21	0.06
	3b Communication culture in the team / in the enterprise	2.96	0.75	3.55	0.60	3.47	0.64	3.58	0.54	3.38	0.69	***0.40	***0.35	***0.43	0.06	0.01	0.07
	3c Leadership culture	2.73	0.81	3.33	0.72	3.31	0.74	3.31	0.74	3.15	0.80	***0.36	***0.34	***0.35	0.01	0.01	0.00
	3d Leadership quality of superiors	3.26	0.75	3.68	0.55	3.63	0.54	3.65	0.56	3.54	0.64	***0.30	***0.26	***0.28	0.06	0.03	0.03
4 Physical work environment (item)	Physical work environment	2.45	0.68	2.37	0.73	2.72	0.82	2.77	0.75	2.59	0.76	0.08	*0.16	***0.21	***0.22	***0.27	0.04
5 Individual disposition (item)	Employees' individual disposition	3.21	0.66	3.29	0.59	3.32	0.51	3.06	0.55	3.21	0.59	0.05	0.06	**0.14	0.01	***0.20	***0.23

Note. Abbreviations: HRM= Human Resource Managers (n = 176-170), OP= Occupational Physicians (n = 127-133), PCP= Primary Care Physicians (n = 133-136 , PT= Psychotherapists (n = 178-184) , (n = 611-622), M = mean, SD = standard deviation, significant $p_{(Wilcoxen)}$ = *p < 0.05, **p < 0.01, ***p < 0.001, w = effect size of differences, Mean values of 4-point-Likert-scaled answers from 1 (not important at all) to 4 (very important).
